# A 5-min paradigm to evoke robust emotional reactivity in neuroimaging studies

**DOI:** 10.3389/fnins.2023.1102213

**Published:** 2023-03-07

**Authors:** Dean Sabatinelli, Constantin Winker, Andrew H. Farkas, Maimu A. Rehbein, Markus Junghoefer

**Affiliations:** ^1^Department of Psychology and Neuroscience, BioImaging Research Center, University of Georgia, Athens, GA, United States; ^2^Institute for Biomagnetism and Biosignal Analysis, University of Münster, Münster, Germany; ^3^Otto Creutzfeldt Center for Cognitive and Behavioral Neuroscience, University of Münster, Münster, Germany

**Keywords:** RDoC, emotion, functional MRI, protocol, stimuli, amygdala, visual perception

## Abstract

The advent of the Research Domain Criteria (RDoC) approach to funding translational neuroscience has highlighted a need for research that includes measures across multiple task types. However, the duration of any given experiment is quite limited, particularly in neuroimaging contexts, and therefore robust estimates of multiple behavioral domains are often difficult to achieve. Here we offer a “turn-key” emotion-evoking paradigm suitable for neuroimaging experiments that demonstrates strong effect sizes across widespread cortical and subcortical structures. This short series could be easily added to existing fMRI protocols, and yield a reliable estimate of emotional reactivity to complement research in other behavioral domains. This experimental adjunct could be used to enable an initial comparison of emotional modulation with the primary behavioral focus of an investigator’s work, and potentially identify new relationships between domains of behavior that have not previously been recognized.

## Introduction

The advent of the Research Domain Criteria (RDoC) approach to funding translational neuroscience has highlighted a need for research that includes measures across multiple task types (Insel et al., 2010; Morris and Cuthbert, 2012). This manifold approach, put forth by the National Institute of Mental Health (NIMH) has led to novel advances in our understanding of complex mental disorders such as depression (Drysdale et al., 2017), anxiety (McTeague and Lang, 2012), and psychosis (Clementz et al., 2016). The majority of mental disorders include substantial emotional symptoms (American Psychiatric Association., 2022), and one major theme of the RDoC approach is to improve the utility of individual studies of mental disorders by expanding data collection across behavioral domains, including cognitive, social, and affective processing. However, the duration of any given experiment is limited (particularly in neuroimaging contexts), and robust estimates of multiple behavioral domains are often difficult to achieve. There is a need for time-efficient paradigms to evoke emotion states with robust measurement properties that can be easily integrated into diverse research programs (LeDoux and Pine, 2016; Peng et al., 2021). Here we offer a brief yet robust emotional perception paradigm suitable for neuroimaging experiments that evokes strong activation effect sizes in cortical and subcortical structures known to be engaged by emotional processing. This 5-min series could be added to any fMRI protocol, and yield a reliable estimate of emotional reactivity to complement research in other behavioral domains.

Naturalistic scene stimuli are often used to evoke emotion in human neuroscience studies. This approach has led to considerable progress in our understanding of basic and translational research problems in psychology and psychiatry (McTeague et al., 2012; Bradley et al., 2014). The majority of neuroimaging investigations of emotional perception have identified modulation of brain activity evoked by naturalistic scenes that can be classified according to two primary dimensions of valence (pleasantness) and arousal. A reliable finding is that emotionally arousing scenes, whether pleasant or unpleasant, evoke enhanced activation in a broad network of regions, including the visual system, amygdala, thalamus, anterior insula, and ventral prefrontal cortex (Kober et al., 2008; Liu et al., 2011; Sabatinelli et al., 2011; Sabatinelli and Frank, 2019). The utility of the emotional scene paradigm for the study of psychosis within the RDoC framework is evident in recent research of emotional scene perception, which altered emotional reactivity in bipolar disorder with psychosis as compared to healthy controls (Trotti et al., 2020), and blunted emotional reactivity in schizophrenia and schizoaffective disorder (Trotti et al., 2021).

The novel purpose of this report is to offer an opportunity for “cross-fertilization” of BOLD signal patterns in emotion-sensitive brain networks with BOLD signal patterns evoked in another behavioral domains that may have relevance to defining the mechanisms of mental disorders. The paradigm described here will provide neuroimaging researchers with a straightforward means to add an assessment of emotional reactivity to any study. The basic turn-key package will include the scene stimuli and the Python code to present the scenes in an event-related fashion in under 5 min. Brain activation magnitude and emotional effect sizes are described based on an unselected sample of 23 healthy participants, using common acquisition, reduction and analysis procedures, but the parameters of image collection and analysis are open to the needs and preferences of the researcher.

## Methods

### Participants

Our intention is provide a credible demonstration of the emotional impact that a researcher might expect when using a sample size that is typical of human fMRI studies (Szucs and Ioannidis, 2017). Twenty-four members of the University of Münster community participated in the fMRI experiment. All participants gave written informed consent, as approved by the University of Münster Human Subjects Review Board. One participant’s data was excluded due to excessive head motion, leaving 23 participants in the final sample (11 female, mean age 26.9 years, SD 3.8 years). All participants reported no neurological abnormalities and had normal or corrected-to-normal vision. Participants received 50 Euros compensation.

### Experimental procedure

In brief, the functional imaging protocol involves a passive scene viewing period of 5 min in the scanner. Participants are asked to maintain central fixation on a projection screen while the scene stimuli are presented in a mixed, event-related design.

Participants were fitted with earplugs, headphones, and given a patient-alarm squeeze ball prior to entering the bore of the scanner. Padding inside the head coil and explicit verbal instruction were used to limit head motion. After a 5-min structural volume was collected, a 5-min functional scan was collected. Participants were instructed to attend to the series of 20 emotional and neutral scenes, while maintaining fixation on a red fixation dot at the center of the screen. The scenes were rear-projected onto a screen visible through a coil-mounted mirror. Following the 20 scene series, participants left the scanner room and reviewed each scene on a laptop computer, while providing their ratings of pleasantness and emotional arousal using the Self-Assessment Manikin (SAM; Bradley and Lang, 1994). Participants then received transcranial direct current stimulation, and viewed two additional scene series while functional imaging data was collected (described in Junghöfer et al., 2017).

### Scene stimuli

Participants viewed an event related series of 20 natural scene photographs presented in 256 levels of grayscale, at 800 × 600 resolution, over a 30° horizontal field of view. The stimuli depict 8 pleasant (happy children and families, erotica), 4 neutral (people in daily activities, land, and cityscapes), and 8 unpleasant scenes (threatening people and animals, bodily injuries). All scene stimuli included a central fixation point and were balanced across emotional and neutral content to be statistically equivalent in luminosity and complexity as measured by the 90% quality Joint Photographic Experts Group (JPEG) file size using GIMP 2.8.^1^

The scene series began with a 2 s checkerboard acclimation image, followed by the 20 experimental scenes, presented for 2 s each, in pseudorandom order, separated by interstimulus intervals of 10 or 12 s. The total acquisition time was 4 min and 42 s, including 5 dummy acquisitions (10 s) prior to the first trial to allow MR signal stabilization.

In the original experiment, the scenes were presented using the Psychophysics Toolbox (Brainard, 1997) which runs in Matlab (Mathworks, Inc.). To enable researchers to avoid a dependence on a Matlab license, the presentation paradigm was rewritten using open-source Psychopy software (Peirce et al., 2019). The code was created with the “builder” graphic user interface such that it can be simple to use and adjust for individual research applications. The code includes a generic trigger wait loop to enable synchronization with the first functional image acquisition, which will need to be set appropriately for each scanner and chosen image repeat time (the default is a 2 s TR). In addition, the scene presentation order is randomized for each run, with a requirement that a specific scene content (e.g., pleasant, neutral, unpleasant) is not repeated twice in succession. This process occurs before the experiment is fully loaded. There is also a means of tracking and correcting the slight time variation (∼1–2 ms) that occurs as each scene is loaded into video memory. This variation is corrected in the following inter trial interval, such that each trial has the same duration, and the total presentation series ends on time. The scene presentation order and onset/offset times are recorded in a log file for each run.

### MR data acquisition and reduction

Using a Siemens Prisma 3T MR scanner (Siemens Healthcare, Erlangen, Germany), a T1-weighted structural volume was collected consisting of 192 sagittal slices with a 256 × 256 matrix and 1 mm^3^ isotropic voxels. The functional prescription covered the whole brain with 30 interleaved axial slices with 3.5 mm^3^ isotropic voxels (64 × 64 gradient echo EPI, 224 mm FOV, 3.5 mm thickness, no gap, 90° flip angle, 30 ms TE, 2000 ms TR). The functional time series was slice-time corrected using cubic spline interpolation, spatially smoothed across 2 voxels (7 mm full width at half maximum), linearly detrended and high-pass filtered at 0.02 Hz. Structural and co-registered functional data were resampled into 1 mm^3^ voxels and transformed into standardized Talairach coordinate space, using BrainVoyager (Brain Innovation).^2^

### Estimates of BOLD signal reactivity

A repeated measures ANOVA using a 2-gamma hemodynamic response function (Friston et al., 1998) convolved with scene presentations assessed the effect of emotional scene content on brain activation. The resulting group-averaged BOLD signal map was thresholded at a false discovery rate of *p* < 0.01 (Genovese et al., 2002) and a minimum cluster size of 250 mm^3^. Our goal in this paper was to enhance the reliability of our BOLD activation results, thus we chose a relatively large ROI volume to enable the averaging of roughly 70 voxels at raw acquisition size. From each participant’s data, 5 regions of interest (ROI) were sampled, the locations of which were derived from prior studies employing this paradigm (Frank and Sabatinelli, 2014, 2019; Sambuco et al., 2020) as well as 3 large meta-analyses of visual emotional processing (Kober et al., 2008; Liu et al., 2011; Sabatinelli et al., 2011). These ROIs are not intended to represent an complete list of all brain regions that show emotional sensitivity, but instead are used to demonstrate the effects sizes of BOLD signal differences that may be expected when employing this paradigm and protocol. These regions include bilateral amygdala (±22, −7, −12), fusiform gyrus (FG; ±44, −50, −14), lateral occipital cortex (LOC; ±45, −70, 2), inferior frontal gyrus/anterior insula (IFG; ±34, 19, 6), and the medial dorsal nucleus of the thalamus (MDN; 0, −19, 5). A 100 mm^3^ sample of BOLD signal was extracted from each ROI location for each participant. From these functional time series, BOLD signal change scores were calculated using the average percent signal change 4–8 s following scene onset, deviated from the 2 s pre-stimulus baseline.

## Results

### Scene content effects

**FIGURE 1:**
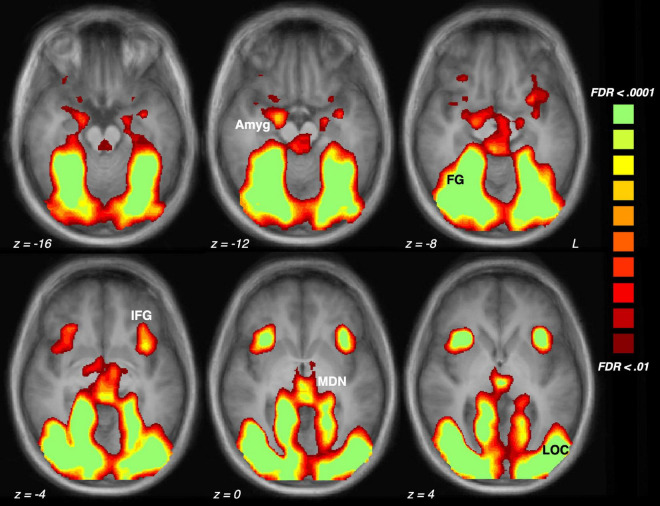
Brain activation driven by emotional (pleasant and unpleasant) compared to neutral scene perception across the sample of 23 participants. The five regions of interest are indicated: Amyg, amygdala; FG, fusiform gyrus; IFG, inferior frontal gyrus/anterior insula; MDN, medial dorsal nucleus of the thalamus; LOC, lateral occipital cortex.

**FIGURE 2:**
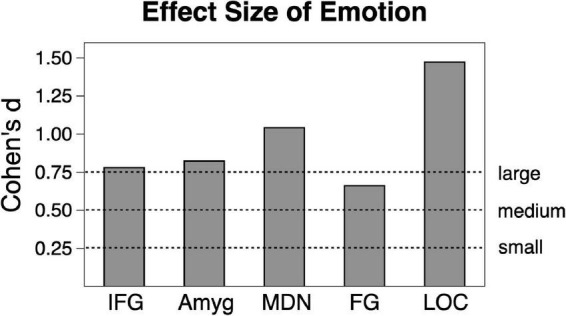
Emotional vs. neutral contrast Cohen’s effect size across the sample (*n* = 23) in the five regions of interest. Amyg, amygdala; FG, fusiform gyrus; IFG, inferior frontal gyrus/anterior insula; MDN, medial dorsal nucleus of the thalamus; LOC, lateral occipital cortex.

Analysis of scene content effects (Figure 1) were assessed with repeated measures ANOVA across pleasant, neutral and unpleasant scenes, applying corrections for violations of sphericity where necessary. Effect sizes will be listed in generalized η^2^ (where 0.01 indicates a small effect, 0.06 indicates a medium effect, and 0.14 indicates a large effect) and in Cohen’s d for ease of interpretation in Figure 2.

As expected, scene content significantly modulated ratings of valence (*F*(2,44) = 168.71, *p* < 0.001, *η_*G*_^2^* = 0.818) and ratings of arousal (*F*(2,44) = 99.86, *p* < 0.001, *η_*G*_^2^* = 0.741). On scales of 1–9 (standard error), the sample rated pleasant scenes at 7.12 (0.181), followed by neutral scenes at 6.70 (0.184), followed by unpleasant scenes at 2.99 (0.175). On a scale of 1–9, the sample rated unpleasant scenes as most arousing at 6.90 (0.196) followed by pleasant scenes at 5.53 (0.203) and neutral scenes at 2.87 (0.235).

**FIGURE 3:**
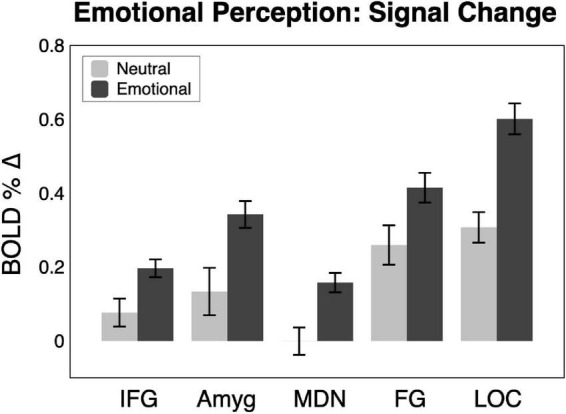
BOLD signal change across the sample (*n* = 23) in the five regions of interest for neutral and emotional scenes. Amyg, amygdala; FG, fusiform gyrus; IFG, inferior frontal gyrus/anterior insula; MDN, medial dorsal nucleus of the thalamus; LOC, lateral occipital cortex.

Shown in Figures 2, 3, emotional scene perception significantly modulated BOLD signal in amygdala (*F*(2,44) = 6.34, *p* < 0.01, *η_*G*_^2^* = 0.138), fusiform gyrus (*F*(2,44) = 12.94, *p* < 0.001, *η_*G*_^2^* = 0.129), lateral occipital cortex (*F*(2,44) = 30.10, *p* < 0.001, *η_*G*_^2^* = 0.337), inferior frontal gyrus/anterior insula (*F*(2,44) = 5.47, *p* < 0.01, *η_*G*_^2^* = 0.127), and medial dorsal nucleus of the thalamus (*F*(2,44) = 5.83, *p* < 0.01, *η_*G*_^2^* = 0.172).

Consistent with much prior work, both pleasant and unpleasant scenes elevated BOLD signal compared to neutral scene perception in all regions. To provide an estimate of the strength of emotional reactivity in each ROI, Cohen’s d effect size was calculated for BOLD signal enhancement by emotional (pleasant and unpleasant) relative to neutral scene perception, as shown in Figure 2. These effect sizes were quite strong, ranging from medium to large in FG (0.66), large in IFG (0.78), large in amygdala (0.82), large in MDN (1.04), and very large in LOC (1.47).

## Discussion

Scientific progress is often produced through the study of a single domain of behavior in a simplified model. The opposing trend toward interdisciplinary and multimodal approaches toward longstanding research questions has been formalized in the RDoC funding initiative for mental health research. The use of multiple measures that cut across multiple behavioral domains is desirable, but any single experiment is of course limited in scope and duration. Here we offer a 5-min turn-key paradigm that can be added to an fMRI protocol that can evoke substantial emotional reactivity across widespread regions of the brain. This short extension would allow investigators to assess the potential relationships between the dependent measures of their target behavioral domain to emotional modulation based on scene perception, without need for a local collaborator, and with predictable effect sizes, even in a relatively small sample. The emotional scenes as well as the open-source Python code to present those is freely available on the Open Science Framework.^3^ The investigator will only need to insert the necessary information to register the first RF trigger of the scanner to time lock scene presentation to image acquisition.

The naturalistic emotional scene paradigm has been employed widely and provides several benefits as compared to other emotion-elicitation paradigms. No task training is required, no specific language or culture is represented, and the scenes are inherently engaging. There is no dependence on the interpretation of facial expressions, which can conflate socio-communicative and emotional processes (Barrett et al., 2019), and are known to differentially impact clinical and non-clinical populations (Anticevic et al., 2012; Filkowski and Haas, 2016).

We consider this brief paradigm to represent a robust, but basic assessment of emotional reactivity, that recruits a broad network of structures shown repeatedly to be active in emotional perception (Sabatinelli et al., 2011; Sambuco et al., 2020). The 5-min series is intended to be an adjunct to an existing fMRI protocol, and serves to provide a predictable index of emotional network activity. This index could be used in an exploratory manner to enable an initial comparison of emotional modulation with the primary behavioral focus of an investigator’s work. In this minimal form, interpretive limitations are understood to be compromises of time considerations, and should potentially meaningful relationships between the primary behavior of interest and emotional reactivity arise, a more extended assessment of emotional reactivity would be justified. For example, predictions regarding the relative impact of pleasant and unpleasant scenes are not sufficiently powered in this 5-min protocol, due to the fact that the great majority of brain reactivity is driven by emotional arousal, and not valence (Kober et al., 2008; Liu et al., 2011; Lindquist et al., 2016). If an investigator can afford more time in the scanner, and desires more trials and scene variability, the International Affective Picture System (Lang et al., 2008) is widely used, or additional perceptually balanced scene sets are available from the corresponding author, depicting a broad range of contents.

## Data availability statement

The raw data supporting the conclusions of this article will be made available by the authors, without undue reservation.

## Ethics statement

The studies involving human participants were reviewed and approved by the University of Münster Human Subjects Review Board. The patients/participants provided their written informed consent to participate in this study.

## Author contributions

DS and MJ designed, conducted, analyzed the data and wrote the manuscript for this study. MR and CW conducted, analyzed the data and reviewed the manuscript for this study. AF analyzed the data and wrote the presentation code for the dissemination of the turn-key study protocol. All authors contributed to the article and approved the submitted version.

## Footnotes

1
https://www.gimp.org

2
https://brainvoyager.com/

3
https://osf.io/57xgk/
